# Perforated Meckel’s Diverticulum in an Infant: A Case Report and Review of the Literature

**DOI:** 10.7759/cureus.85066

**Published:** 2025-05-29

**Authors:** Kostas Tepelenis, Maria Alexandra Kefala, Margarita Efthalia Papasavva, Aikaterini Gkrepi, Vasiliki Tsanaka, Vasilios Grammeniatis, Konstantina Georgopoulou, Gerasimia D Kyrochristou, Ilektra Kyrochristou, Vasiliki Gketsi

**Affiliations:** 1 Surgery, General Hospital of Ioannina G. Hatzikosta, Ioannina, GRC; 2 Pediatrics, General Hospital of Ioannina G. Hatzikosta, Ioannina, GRC; 3 Surgery, University General Hospital of Ioannina, Ioannina, GRC; 4 Medicine, School of Health Sciences, University of Ioannina, Ioannina, GRC

**Keywords:** infant, intestinal perforation, meckel´s diverticulum, perforated meckel's diverticulum, peritonitis

## Abstract

Meckel’s diverticulum (MD) is the most common congenital malformation of the gastrointestinal tract, but perforation of this diverticulum is a rare complication, especially in infants. Diagnosing this complication before surgery can be difficult, and it is typically confirmed through laparotomy. This report describes an eight-month-old female infant who was brought to the emergency department with a history of persistent crying, fever, vomiting, and one occurrence of currant jelly stool in the past 12 hours. A physical examination revealed abdominal distension, tenderness, and rigidity. An abdominal ultrasound indicated free intra-abdominal fluid containing micro-echoic elements. An exploratory laparotomy was performed, during which pus was found in the abdominal cavity. A perforated MD was identified, located 60 cm proximal to the ileocecal valve. An enterectomy and end-to-end anastomosis were performed to remove the perforated diverticulum. The postoperative course was uneventful, and the infant was discharged on the eighth postoperative day. Histopathological examination confirmed the presence of a perforated MD with ectopic gastric mucosa. This report summarizes and characterizes the presentation, diagnosis, and management of this rare complication.

## Introduction

Fabricius Hildamus first identified an anatomical variant in the ileum in 1598 but did not recognize its embryological origin. The German anatomist Johan Friedrich Meckel later described Meckel’s diverticulum (MD) in 1809 as a remnant of the omphalomesenteric duct [1]. MD is the most common congenital anatomical malformation of the gastrointestinal tract [2]. It results from the omphalomesenteric duct, which typically obliterates during the fifth to seventh week of gestation [3]. MD is usually located on the anti-mesenteric border of the ileum, approximately 50-75 cm from the ileocecal valve [4, 5]. Anatomically, it is classified as a true diverticulum because it contains all layers of the small intestine [6] and may contain various types of ectopic tissue, including gastric, pancreatic, colonic, duodenal, or endometrial tissue, in 30%-50% of cases [7].

The estimated incidence of MD is about 2%. It most commonly affects children around the age of 2, with a higher prevalence in males, at a ratio of 2:1. MD is classically defined by the *rule of twos*: (1) it occurs in 2% of the population, (2) there is a male predisposition with a ratio of 2:1, (3) the age of presentation is typically around two years, (4) it is usually located within 2 feet from the ileocecal valve, and (5) it is generally about 2 inches in length [6,8]. Most cases of MD are asymptomatic and are often discovered incidentally. The lifetime risk of complications is approximately 4%, which may include rectal bleeding, intussusception, intestinal obstruction, and diverticulitis [5]. Perforation occurs less frequently, accounting for about 10% of cases [9].

Diagnosing a perforation of MD before surgery can be challenging because specific imaging findings are often absent; typically, the diagnosis is confirmed during laparotomy. In neonates, perforation due to MD is rare, with only a few case reports in the literature [6]. However, such cases are even less common in infants. The first documented instance involved a 13-month-old boy who died shortly after being admitted to the hospital, where an autopsy revealed a perforation of the MD. [5]. In this report, we discuss the case of an eight-month-old girl who experienced perforation of MD and subsequently underwent intestinal resection followed by end-to-end anastomosis.

## Case presentation

An eight-month-old female infant, previously healthy, presented to the emergency department with persistent crying, fever, vomiting, and one episode of currant jelly stool over the past 12 hours. The highest recorded temperature was 38 °C. Her parents also reported an earlier episode of currant jelly stool two days prior. 

**Figure 1:**
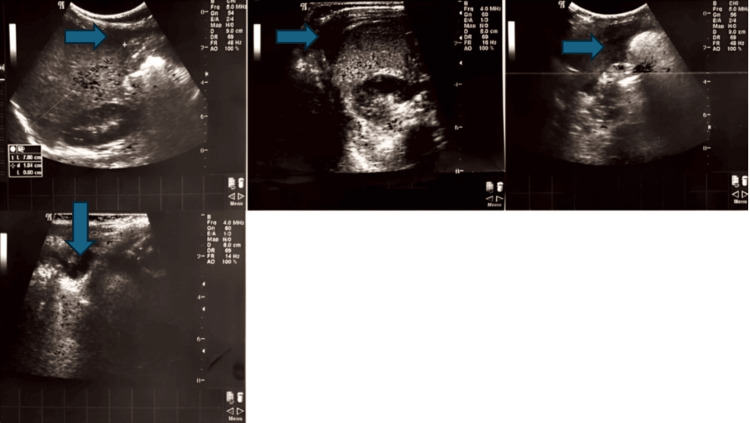
Free intra-abdominal fluid containing micro-echoic elements (blue arrows).

On examination, her vital signs were as follows: blood pressure 96/73 mmHg, heart rate 180 beats per minute, respiratory rate 24 breaths per minute, and a temperature of 37.8 °C. Physical examination revealed abdominal distension with tenderness and rigidity, accompanied by absent bowel sounds. Laboratory tests yielded elevated erythrocyte sedimentation rate (17 mm/hour), normal white blood cell count (9.91 k/μL) with 45.5% neutrophils and 51.3% lymphocytes, normal CRP (<0.1 mg/dL), hematocrit 30.5%, hemoglobin 9.8 g/dL, and platelets 157 k/μL (Table 1). An abdominal ultrasound revealed free intra-abdominal fluid, with the peritoneal fluid showing micro-echoic elements (Figure 1). 

**Table 1:** Laboratory test results. WBC, white blood cell; NEUT, neutrophil; LYMPH, lymphocyte; MONO, monocyte; EO, eosinophil; BASO, basophil; RBC, red blood cell; HGB, hemoglobin; HCT, hematocrit; MCV, mean corpuscular volume; MCH, mean corpuscular hemoglobin; MCHC, mean corpuscular hemoglobin concentration; PLT, platelet; ESR, erythrocyte sedimentation rate; GLC, glucose; URE, urea; CRE, creatinine; K+, potassium; Na+, sodium; CL, chloride; TPR, total protein; ALB, albumin; ALP, alkaline phosphatase; AST, aspartate aminotransferase; ALT, alanine aminotransferase; γGT, gamma-glutamyl transferase; LDH, lactate dehydrogenase; CK, creatine kinase; AMY, amylase; LIP, lipase; CA^++^, calcium; PO4^---^, phosphate; CRP, C-reactive protein

Test	Result	Reference range
WBC	9.91 k/μL	4-11 k/μL
NEUT	45.5%	40%-75%
LYMPH	51.3%	20%-45%
MONO	2.8%	2%-10%
EO	0.2%	1%-6%
BASO	0.2%	0.2%-1%
RBC	4.16 M/μL	3.8-6 M/ μL
HGB	9.8 g/dL	11.8-17.8 g/dL
HCT	30.5%	36%-52%
MCV	73.3 fL	80-96 fL
MCH	23.6 pg	26-32 pg
MCHC	32.1 g/dL	32-36 g/dL
PLT	157 k/μL	140-450 k/μL
ESR	17 mm/hour	<10 mm/hour
GLC	273 mg/dL	70-125 mg/dL
URE	30 mg/dL	11-54 mg/dL
CRE	0.49 mg/dL	0.5-1.4 mg/dL
K+	4.1 mmol/L	3.5-5.1 mmol/L
Na^+^	136 mmol/L	136-146 mmol/L
CL	103 mmol/L	98-106 mmol/L
TPR	6.1 g/dL	6.4-8.3 g/dL
ALB	4.2 g/dL	3.5-5 g/dL
ALP	228 IU/L	35-125 IU/L
AST	37 U/L	5-40 U/L
ALT	11 IU/L	5-40 IU/L
γGT	7 IU/L	8-35 IU/L
LDH	218 IU/L	120-230 IU/L
CK	159 IU/L	0-220 IU/L
AMY	33 IU/L	28-100 IU/L
LIP	40 U/L	0-35 U/L
CA^++^	9.7 mg/dL	8.2-10.6 mg/dL
PO4^---^	5.4 mg/dL	4-7 mg/dL
CRP	<0.100 mg/dL	0.00-0.800 mg/dL

**Figure 2:**
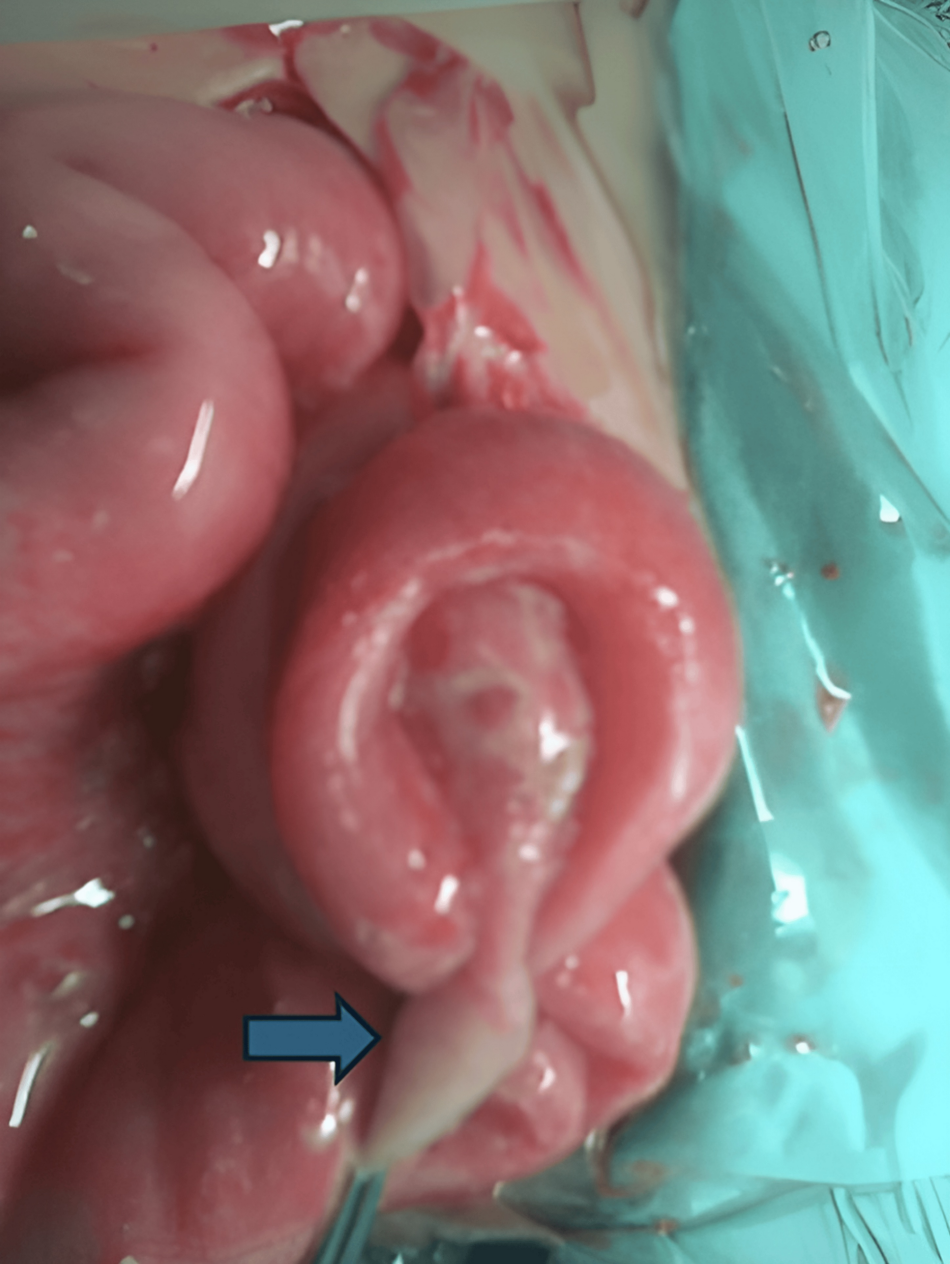
A Meckel's diverticulum was identified 60 cm proximal to the ileocecal valve, with the tip of the diverticulum showing perforation (blue arrow).

The consulting surgical team performed an exploratory laparotomy. At the beginning of the procedure, a significant amount of turbid pus was observed leaking from the abdominal cavity. An MD was identified 60 cm proximal to the ileocecal valve, with the tip of the diverticulum showing perforation (Figure 2). An enterectomy, including the removal of the MD, was performed, followed by an end-to-end anastomosis. The postoperative period was uneventful; the infant began oral intake on the third postoperative day and was discharged on the eighth postoperative day. The histological report confirmed a perforated MD with ectopic gastric mucosa.

## Discussion

The German anatomist Johan Friedrich Meckel first identified MD as a remnant of the omphalomesenteric duct in 1809. However, earlier mentions of it as an anatomical variant in the ileum were made by Fabricius Hildamus in 1598 and Lavater in 1671, both of whom did not recognize its embryological origin [1]. MD is the most common congenital anatomical malformation of the gastrointestinal tract [2]. During fetal development, the omphalomesenteric duct connects the yolk sac to the intestinal tract and typically disappears between the fifth and seventh week of gestation. Occasionally, the omphalomesenteric duct fails to obliterate, resulting in congenital anomalies such as residual fibrous cords, umbilical sinus, omphalomesenteric fistula, enterocyst, and, most commonly, MD [1,3].

MD is a small pouch that typically measures between 3 and 6 cm in length, generally protruding from the antimesenteric side of the ileum, around 50 to 75 cm away from the ileocecal valve [4-6]. In terms of its anatomy, it is categorized as a true diverticulum because it includes all the layers of the small intestine [6]. In 30% to 50% of cases, MD contains ectopic tissue, which can include gastric tissue (60%), pancreatic tissue (6%), duodenal tissue (2%), and jejunal tissue (2%) [7]. The blood supply to MD is typically provided by the omphalomesenteric artery, which arises from an ileal branch of the superior mesenteric artery [10]. The incidence of MD is approximately 2%, and it can present at any age, though it most often affects children around two years old and shows a male predisposition. This condition typically adheres to the *rule of twos*: (1) it affects around 2% of the population, (2) there is a higher prevalence in males, with the male-to-female ratio of 2:1, (3) it typically presents around the age of two years, (4) it is commonly found within 2 feet of the ileocecal valve, and (5) its average length is about 2 inches [6,8].

MD is often discovered incidentally, as it is frequently asymptomatic. The complication rate is low, estimated at around 4% [5,11]. Yamaguchi et al. reviewed 287 symptomatic patients and found that the most common complication was intestinal obstruction (36.5%), followed by intussusception (13.7%), diverticulitis (12.7%), rectal bleeding (11.8%), perforation (7.3%), Littre hernia (4.7%), volvulus (3.2%), neoplasm (3.2%), and umbilical fistula (1.7%) [12]. In another study by St-Vil et al., which analyzed 117 symptomatic patients, 42% presented with bowel obstruction, 38% had rectal bleeding, 14% had diverticulitis, and 6% experienced umbilical pathology. The primary causes of bowel obstruction were found to be volvulus (40.8%) and intussusception (38.8%) [13]. Conversely, Lee et al. reviewed 58 symptomatic patients and reported rectal bleeding as the most typical clinical manifestation (76%), followed by intestinal obstruction (34%), perforation (13%), and diverticulitis (9%). The leading causes of intestinal obstruction in this group were intussusception (38%) and internal hernia (27%) [14].

Perforation of MD occurs less commonly, accounting for about 10% of cases [5,9-11]. The exact cause of this condition remains unclear, and several theories have been proposed. One theory suggests that ulceration of the adjacent ileal mucosa, due to acid produced by ectopic gastric tissue, can lead to perforation. Another possibility is that a progression of diverticulitis could cause erosion of the intestinal wall, ultimately resulting in perforation. Additionally, the knotting of a long MD around itself may create weakness in the intestinal wall, leading to perforation [5,6]. Some authors also propose that perforation could result from separating vitelline remnants from the abdominal wall. Furthermore, ingested foreign bodies, such as fish or chicken bones and bay leaves, might damage the intestinal wall and cause perforation [5]. In the current case, an ectopic gastric mucosa was found on pathology.

Preoperative diagnosis of a perforated MD is challenging due to the lack of specific symptoms, signs, and imaging findings. It typically manifests with symptoms of acute abdomen and abdominal distention, while an X-ray can reveal the presence of intraabdominal free air [6,9]. A definitive diagnosis is usually established during laparotomy. In terms of management, the preferred treatment for a perforated MD is resection followed by end-to-end anastomosis [6].

Several cases of MD perforation have been reported in neonates, although such occurrences are extremely rare in infants and can be challenging to diagnose [6]. The first documented case of perforated MD in a 13-month-old male infant was presented by Türkmen et al. in 2013. Unfortunately, the infant passed away shortly after being admitted to the hospital. An autopsy examination revealed a foul-smelling fluid that was cream-brown and greenish, filling the abdomen. A perforated MD, measuring 2 x 2 cm, was located 45 cm proximal to the ileocecal valve [15].

In another case described by Lee et al., a 13-month-old male infant arrived at the emergency department with currant jelly stools lasting approximately 24 hours. Initially, intussusception or bacterial enteritis was suspected. However, an abdominal ultrasound showed no signs of intussusception or appendicitis. On the third day of hospitalization, the infant developed a high fever and irritability. An abdominal computed tomography scan was performed, revealing an intraperitoneal and retroperitoneal abscess with air collection, likely due to bowel perforation. The infant underwent exploratory laparotomy, which showed a 5 cm MD with a perforation at the tip located 50 cm proximal to the ileocecal valve. A diverticulectomy and bowel wall repair were performed. The postoperative recovery was uneventful, and the infant was discharged on the fifth postoperative day [5].

Lastly, Zhou et al. reported the case of a seven-month-old male who was admitted to the hospital after experiencing persistent crying and fever for two days. An abdominal computed tomography scan indicated slight expansion with gas and fluid accumulation in some intestinal loops, along with a small amount of free fluid in the abdomen. On the second day of hospitalization, the infant continued to have recurrent fever, signs of infection, and localized peritoneal irritation. An exploratory laparotomy was conducted, revealing a perforated MD approximately 30 cm from the ileocecal valve. Diverticulectomy and repair of the bowel wall were performed. The postoperative recovery was also uneventful; oral intake began on the third postoperative day, and the infant was discharged on the tenth postoperative day. The pathology report indicated chronic diverticulitis with ectopic pancreatic mucosa [16].

## Conclusions

MD is the most common congenital anatomical malformation of the gastrointestinal tract; however, its perforation in infants is extremely rare. Diagnosing perforated MD before surgery can be challenging due to the absence of specific symptoms, signs, and imaging findings. When infants present with unexplained acute abdominal pain, it is important to maintain a high suspicion for perforated MD. Taking prompt action, such as performing a laparotomy or laparoscopy, is crucial to prevent complications and to confirm the diagnosis.
